# Taking medical humanities forward

**DOI:** 10.3352/jeehp.2011.8.7

**Published:** 2011-07-27

**Authors:** P. Ravi Shankar, Rano M Piryani

**Affiliations:** 1 Department of Medical Education, KIST Medical College, Lalitpur, Nepal.; 2 Department of Medicine, KIST Medical College, Lalitpur, Nepal.

Dear editor

We read with great interest the article titled 'Reaching people through medical humanities: an initiative' published in your journal [1]. The authors have formed a Medical Humanities group in their institution and have organized lectures and plays on topics related to medical humanities (MH). We are also happy to note the positive response towards the discipline and the authors' plans for the future.

In South Asia a region with a large proportion of the world's medical schools and population MH programs are uncommon. We had conducted a voluntary module at Manipal College of Medical Sciences, Pokhara [2] and are at present conducting a module for first year students at KIST Medical College (KISTMC), Lalitpur, Nepal [3]. Our program is running well and certain other schools have shown interest in starting MH programs. MH has a number of advantages in the education of doctors. On searching the literature using PubMed, Google, and Google Scholar we were not able to come across literature dealing with other MH programs in South Asia. Initiatives are slowly being taken but a formal module is yet to be initiated to the best of our knowledge.

We think the next step for the authors would be to start a MH session using small group activity-based learning. Painting, literature, sculpture, drama, films and other modalities can be used to explore the humanities. Case scenarios, role-plays and debates can be interesting learning modalities. The group already has interested and motivated faculty who can act as facilitators. In Delhi, the authors may have the advantage of close interaction with and support from humanities teachers in other colleges. Access to paintings and literature will also be easy. The authors can take steps to ensure that their college library stocks a good number of general books in addition to medical textbooks. The authors have laid the ground for MH through the 'Confluence' series and this has to been built upon. The authors' experiences with a MH module will be of special interest to us in Nepal, another South Asian country. South Asian experiences will help create a body of knowledge which will be of help to those interested in starting modules in their institution. We wish them all the best in this endeavor.
